# Safety, tolerability and pharmacokinetics of DNDI‐6148, a novel agent for leishmaniasis: A randomized, controlled, single ascending dose study in healthy participants

**DOI:** 10.1002/bcp.70328

**Published:** 2025-11-20

**Authors:** Jean‐Yves Gillon, Sophie Delhomme, Delphine Launay, Séverine Blesson, Stéphanie Braillard, Pegah Maghdooni, Frauke Assmus, Richard Hoglund, Joel Tarning, Sabrina Loyau, Byron Arana, Mathilde Latreille‐Barbier, Yves Donazzolo

**Affiliations:** ^1^ Drugs for Neglected Diseases initiative (DNDi) Chemin Camille‐Vidart 15 Geneva Switzerland; ^2^ SGS Belgium SA Vieux Chemin du Poète, 10 Wavre Belgium; ^3^ Mahidol Oxford Tropical Medicine Research Unit, Faculty of Tropical Medicine, Mahidol University, Bangkok, Thailand and Centre for Tropical Medicine and Global Health, Nuffield Department of Clinical Medicine University of Oxford Oxford UK; ^4^ PhinC Development 36, rue Victor Basch Massy France; ^5^ Eurofins Optimed SAS 1, rue des Essarts Gières France

**Keywords:** first‐in‐human, leishmaniasis, new chemical entity, pharmacokinetics, safety

## Abstract

**Aim:**

The benzoxaborole derivative DNDI‐6148 is an antiparasitic agent with activity against multiple *Leishmania* protozoan species, including *L. infantum* and *L. donovani* , which cause visceral leishmaniasis. We investigated the safety, tolerability and pharmacokinetics of single oral doses of DNDI‐6148 in a randomized, parallel‐group, placebo‐controlled, first‐in‐human study in 64 healthy participants.

**Methods:**

Eight cohorts of eight participants each were enrolled. DNDI‐6148, formulated as a suspension in ORA‐Sweet® was administered orally as single 10–380 mg doses. Pharmacokinetics (PK) and safety were assessed for four (cohorts receiving 10–80 mg DNDI‐6148) or six (cohorts receiving 160–380 mg DNDI‐6148) days after dosing.

**Results:**

Sixteen adverse events (AEs) were experienced by 13 participants (20.3%), all mild or moderate in severity and resolved by the end of the study. No AE led to any participant withdrawal, and no fatal or serious AEs were reported. DNDI‐6148 was relatively slowly absorbed (t_max_ ranging between 3 and 12 h) under fasting conditions, with increase in plasma concentrations modestly sub‐proportional to dose. The mean half‐life was between 12.80 and 25.42 h. The fraction of the dose excreted by the kidneys as unchanged DNDI‐6148 was below 0.2%. Trace amounts of three metabolites (formed by oxidation, deboronation, dehydrogenation of DNDI‐6148) were detected in plasma.

**Conclusion:**

After a single administration of up to 380 mg by oral route, DNDI‐6148 had an acceptable safety and tolerability profile and a favourable PK profile. These data support further clinical development.

**What is already known about this subject**

- The benzoxaborole derivative DNDI‐6148 acts principally by inhibiting the cleavage and polyadenylation specificity factor (CPSF3) endonuclease in the *Leishmania* parasite.
- In non‐clinical studies, DNDI‐6148 demonstrated robust efficacy in models of leishmaniasis and an acceptable safety profile.
- DNDI‐6148’s pharmaceutical properties are suitable for onward clinical development.

**What this study adds**

- No major safety concerns were identified in 64 healthy male participants exposed to DNDI‐6148 at single doses up to 380 mg, or placebo.
- DNDI‐6148, administered as an oral suspension, had a favourable pharmacokinetic profile.
- Overall, the data support further clinical investigations with multiple doses.

## 1 INTRODUCTION

Leishmaniasis is a spectrum disease caused by protozoan parasites from over 20 *Leishmania* species. It is transmitted to humans through the bite of infected female phlebotomine sandflies. An estimated 700′000 to 1 million new cases occur annually.^1^ It is associated with three main clinical manifestations: cutaneous, mucocutaneous and visceral leishmaniasis. Cutaneous leishmaniasis (CL, consisting of localized skin ulcers) is the most common syndrome^1^, ^2^, ^3^; however, visceral leishmaniasis (VL) is the most severe form and is lethal if left untreated. Typical VL symptoms are fever, weight loss and the enlargement of the spleen and liver.^4^ VL, or kala‐azar, is endemic in three main global regions (South Asia, Eastern Africa and Latin America) and is most prevalent in Brazil, East Africa and in India. An estimated 50′000 to 90′000 new cases of VL occur annually worldwide.^1^ VL is mostly caused by *L. donovani* in Asia and East Africa, where the disease is anthroponotic, and by *L. infantum* in Latin America and the Mediterranean region, where it is zoonotic, with dogs as the main reservoir. In the human host, *Leishmania* are obligate intracellular parasites of the reticulo‐endothelial system that survive and multiply in different macrophage populations.

There has been great progress in the development of VL treatments over the last 15 years. However, the drugs currently available have variable efficacy depending on the regions where they are used, and they have significant limitations, including parenteral administration (except for the oral drug miltefosine), unfavourable cost and limited safety and tolerability.^5^ Innovative, efficacious and safe short‐course treatments that can be deployed in remote areas where VL occurs are urgently needed. Several all‐new treatments with new chemical entities, designed to be safe, effective and easier to manage at the primary healthcare level, are currently in development. Novartis and the Drugs for Neglected Diseases initiative (DNDi) entered in a collaboration to develop LXE408,^6^ a kinetoplastid‐selective proteasome inhibitor with two ongoing phase 2 studies in VL patients in India and Ethiopia. Also, DNDI‐6899, a pyrazolopyrimidine derivative active against parasitic cdc‐2‐related kinase 12 (CRK12)^7^ is being evaluated in healthy participants in the UK. Moreover, DNDI‐6174^8^ is expected to enter clinical development soon.

DNDI‐6148 is a novel six‐substituted benzoxaborole derivative that is active principally through the inhibition of cleavage and polyadenylation specificity factor (CPSF3) endonuclease, an enzyme involved in the control of polyadenylation and trans‐splicing of pre‐mRNA in the *Leishmania* parasite.^9^ Like several other boron derivatives, DNDI‐6148 has antiparasitic properties.

In vitro, DNDI‐6148 is active against *L. donovani* and *L. infantum* in the micromolar range. DNDI‐6148 demonstrated efficacy by oral route in an *L. infantum* hamster model of VL after 25 mg/kg twice‐daily (BID) dosing for five or ten days and also in an *L. donovani* and *L. infantum* mouse model of VL at the same dose for five days. In addition, DNDI‐6148 is potent in vitro against CL strains with IC_50_ values ranging from 0.05 to 18.3 μM. The unbound fraction of DNDI‐6148 in plasma from mouse, rat, hamster, dog and monkey ranges between 6% and 14% and is 6.6% in humans. Pharmacodynamic properties of DNDI‐6148 appear to be dose‐ and duration‐dependent,^9^ informing human dose prediction and adjustment, though the primary driver of efficacy has not yet been established.

DNDI‐6148 is a promising candidate for an oral, short‐course treatment for both VL and CL. Based on the available non‐clinical data, it has the potential, either as monotherapy or in combination with other anti‐leishmaniasis drugs, to have a substantial curative effect. As part of its clinical development, this first‐in‐human (FIH) phase I trial was conducted to evaluate the safety, tolerability (compared to matching placebo) and pharmacokinetics of single oral doses of DNDI‐6148 in healthy participants.

## 2 METHODS

### 2.1 Ethics

The study was approved (reference: 2–18‐90/SI 1823) by the Comité de Protection des Personnes Sud‐Ouest et Outre‐Mer II, France and was conducted in compliance with the Declaration of Helsinki and the International Conference on Harmonisation E6 Guideline for Good Clinical Practice. Prior clinical trial autorization was obtained from the Agence Nationale de Sécurité du Médicament et des Produits de Santé (ANSM, France). The study was registered in EudraCT (n° 2018–004023‐37) and was performed at Eurofins‐Optimed, Gières, France. Written informed consent was obtained from all participants before undertaking any study‐related procedures. Quality assurance, data management and study monitoring were performed by Eurofins‐Optimed, France.

### 2.2 Trial design

This was a FIH, phase I, double‐blind, parallel, single ascending dose (SAD) study of DNDI‐6148 arginine monohydrate, administered as an oral suspension to healthy male participants. Cohorts 1–8 each included eight participants and were administered 10, 20, 40, 80, 160, 220, 300 or 380 mg (free form) of DNDI‐6148 (in six participants) or placebo (two participants). A sample size calculation was not performed, as six participants per treatment group were deemed sufficient given the exploratory nature of the study.

The initial 10 mg dose was based on the No Adverse Effect Level (NOAEL) in the most sensitive non‐clinical species and sex, the predicted human PK and therapeutic dose, derived from in vitro data on plasma‐protein binding, blood plasma partitioning and from potency and efficacy data obtained in vitro and from studies after administration of DNDI‐6148 to mouse and hamster. Decisions on dose escalation were taken under blinded conditions, based on the safety interim reports and DNDI‐6148 plasma exposure. The trial protocol synopsis is available at https://dndi.org/wp-content/uploads/2023/10/DNDi%E2%80%906148%E2%80%9001-Clinical-Trial-Protocol-Synopsis.pdf

Recruitment was temporarily interrupted on 16 March 2020 due to confinement measures related to the COVID‐19 outbreak in France. The study resumed on 17 August 2020, allowing for the completion of recruitment for cohorts 2, 3 and 4. Additionally, four participants were included in cohort 5 before suspension of the investigational product (IP) administration on 30 January 2021 as a precaution to assess emerging unexpected data in a non‐clinical Fertility and Early Embryonic Development study. The protocol was further amended to include evaluation of plasma concentrations of C‐reactive protein (CRP) and of tumour necrosis factor alpha (TNFα) pre and post‐dose (6 h) as exploratory markers of possible inflammation. Subsequent cohorts were completed up to the 380 mg dose level (cohort 8) without further interruption of the study.

### 2.3 Participants

Men aged 18–50 years with a body mass index of 18.0–30.1 kg/m^2^ were eligible to participate in the study (in line with the limited safety in females in non‐clinical safety assessment). Healthy Caucasian participants were selected at screening based on medical history, physical examination, vital signs, ECG, blood haematology and chemistry, HIV, hepatitis B and C serology and urinalysis. Key exclusion criteria included participation in another clinical study with another investigational product in the past three months, recent use of any prescription medicine, presence or history of severe allergies or blood loss >400 mL. In addition, a negative COVID‐19 test was required at the time of admission to the ward. Full inclusion and exclusion criteria are described in Supplementary Information, Text S1.

### 2.4 Interventions

DNDI‐6148 arginine monohydrate and placebo, developed and manufactured by Syngene (Bangalore, India), were supplied as powder for oral suspension (in bottles of pre‐weighed powder, corresponding to either 60 mg or 600 mg acid‐free equivalent for DNDI‐6148). Manufacturing, packaging, quality control and preparation of clinical supplies complied with Good Manufacturing Practice.

At the clinical site, a suspension was prepared extemporaneously by the pharmacist within 24 h prior to administration to participants (the timepoint for which stability data of the suspension were available). The IP powder was suspended in 30 mL ORA‐Sweet® (Paddock Laboratories, LLC, Minneapolis, USA) for taste and colour masking. Participants were administered between 4 and 25 mL of the suspensions, followed by the ingestion of 250 mL tap water. Opaque precision syringes were used to administer the solutions to minimize the risk of unblinding. To preclude any dietary effects on the PK of DNDI‐6148, participants fasted for 9 h before they received the IP or placebo. Treatments were administered under the supervision of the investigator at approximately 8 a.m. on Day 1.

### 2.5 Randomization and blinding

Randomization was performed by research personnel not involved in the study, using a pre‐determined randomization list and IP allocation. Both participants and study site staff were blinded to the allocation of DNDI‐6148 and matching placebo.

### 2.6 Outcomes

The primary endpoint was assessment of the safety and tolerability of DNDI‐6148 by evaluation of the following parameters: adverse events (AEs), physical examination (including body weight), clinical neurological examination, vital signs, 12‐lead ECG, clinical laboratory (including serum chemistry, haematology, hormonology and urinalysis) and psychological and cognitive examinations.

Secondary pharmacokinetic endpoints for DNDI‐6148 in plasma included C_max_, AUC_0‐∞_, AUC_0–24_, AUC_0‐t_, AUC_0‐t_/D, AUC_0‐∞_/D, C_max_/D, t_max_, t_1/2_, CL/F, Vd/F and %AUC_extra_. Secondary pharmacokinetic endpoints for DNDI‐6148 from urine data included _Ae0‐t_ (t = 24 or 72 h), fe (over 24 h and 72 h) and CL_R_.

Exploratory endpoints included ECG parameters compared to baseline and placebo; the exploratory identification of DNDI‐6148 metabolites; and, for the last four participants of the 160 mg cohort and the 220 mg, 300 mg and 380 mg cohorts, the exploratory evaluation of plasma concentrations of CRP and TNFα pre‐ and post‐dose.

### 2.7 Procedures

Safety was assessed by monitoring AEs throughout the study. Other safety monitoring included 12‐lead ECG recordings, measurement of vital signs, physical and neurological examinations, haematology, blood biochemistry and urinalysis and, for the last four participants of the 160 mg cohort and onwards, CRP and TNFα.

Psychological and cognitive examinations were performed using the Bond & Lader questionnaire and Columbia‐Suicide Severity Rating Scale (C‐SSRS)^10^, ^11^ as DNDI‐6148 was shown in non‐clinical studies to cross the blood–brain barrier (unpublished data). To minimize risk, participants were dosed sequentially, using sentinels in each cohort (one receiving DNDI‐6148, one placebo), as per European Medicines Agency guidelines.^12^ Stopping criteria used in that study are described in Supplementary Information, Text S2. If the previous dose was well tolerated, with no safety concerns, the Safety Review Committee decided on dose escalation after reviewing blinded safety and PK data from all available cohorts.

Blood samples (6 mL per time point) for pharmacokinetic analyses were collected on Day 1 pre‐dose and at the following post‐dose time points: 0.5,1, 1.5 (cohorts 1–4 only), 2, 2.5, 3, 4, 5 (cohorts 5–8 only), 6, 9, 12, 24, 48 and 72 h. Given the half‐life of DNDI‐6148, PK sampling was extended up to 120 h for cohorts 5 and 6 and 168 h for cohorts 7 and 8. Within 30 min of blood collection, blood samples were centrifuged at 1500*g* for 10 min at 4 °C and 500 μL of plasma were transferred into pre‐labelled polypropylene tubes prefilled with phosphoric acid 2% (1:1, v:v). Tubes were capped immediately for each time point, and the plasma was frozen in an upright position at −80 °C for storage.

Urine was collected up to 72 h during the following intervals: pre‐dose, D1T0h – D1T12h, D1T12h – D2T24h, D2T24h – D3T48h and D3T48h – D4T72h. Urine was collected in containers containing 20 g citric acid monohydrate and stored at 4 °C for a maximum of 43 h. All urine passed during each interval period was combined and at the end of the interval, the pool of acidified urine was vigorously mixed; two aliquots were prepared, each with 1 mL of acidified urine and 1 mL of aqueous solution of 50 g/L bovine serum albumin (BSA) and 9 g/L NaCl.

Tubes of acidified urine/BSA samples were capped immediately for each time point and stored at −80 °C. Samples of both acidified plasma and acidified urine/BSA were shipped for bioanalysis on dry ice by a specialized carrier. Temperatures were monitored using a data logger during transport.

### 2.8 PK analyses

Concentrations of DNDI‐6148 were determined in acidified plasma and acidified urine/BSA mixture using a validated internally standardized liquid chromatography–tandem mass spectrometry (LC–MS/MS) assay with deuterated d_4_‐DNDI‐6148 as an internal standard. The lower limit of quantitation (LOQ) for DNDI‐6148 was 1 ng/mL in plasma and 10 ng/mL in urine.

Values that were below the LOQ before the first concentration equal or above LOQ were set to zero. Other values below the LOQ were not used to calculate the PK parameters. The area under the plasma concentration‐time curve from time zero (pre‐dose) to the time of last quantifiable concentration (AUC_0‐last_) was calculated using the linear‐log trapezoidal rule. Elimination half‐life (t_1/2_) was calculated by the equation ln2/λz, after visual inspection of the plasma concentration‐time profiles (considering a minimum of 3 data points, including the last measurable data point and excluding Cmax).

Metabolic profiling of DNDI‐6148 was performed in human acidified plasma samples from cohort 8, which received 380 mg DNDI‐6148. Samples from active participants were pooled by sampling time and analysed using an ultra‐high performance liquid chromatography coupled with mass spectrometry (UHPLC/MS–MS) method (Supplementary Information, Text S3). The putative metabolites were detected and characterized using UHPLC/MS–MS.

### 2.9 Statistical analyses

Statistical analyses were performed using SAS® software version 9.4 (Cary, NC, USA). Demographic data and baseline characteristics were listed and summarized. Safety data did not undergo formal statistical analysis.

PK parameters were derived from plasma concentration *vs*. time data using non‐compartmental analysis in Phoenix WinNonlin® 8.1 (Certara Inc., Princeton, NJ, USA). Plasma concentration *vs*. time data and PK parameters were listed and summarized by dose level, using descriptive statistics. Mean concentrations were calculated only if at least ⅔ of the individual concentrations were above the lower LOQ (LLOQ). Individual participant and mean plasma concentrations were displayed graphically. Planned sampling times were used to summarize plasma concentration data; actual sampling times were used in the derivation of PK parameters. The hypothesis that AUC_0‐∞_ and C_max_ are dose proportional was formally tested using a power model approach based on analysis of variance (ANOVA) techniques.^13^, ^14^

The included set (IS) comprised all randomized participants. The PK set (PKS) included members of the IS without protocol deviations or with violations thought not to significantly affect the pharmacokinetic analysis. The pharmacodynamic set (PDS) included members of the IS without protocol deviations or with violations thought not to significantly affect the pharmacodynamic analysis. The safety set (SS) consisted of members of the IS who received at least one study treatment dose.

## 3 RESULTS

### 3.1 Study population

**FIGURE 1:**
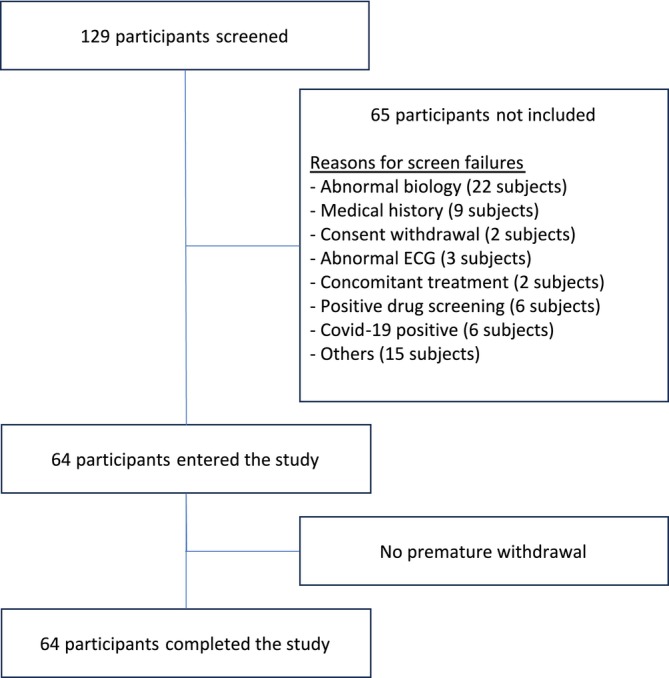
Participant disposition.

After screening 129 participants, enrolment of 64 eligible participants began on 21st January 2020, and the last study visit was on 8th March 2022 (Figure 1). All participants completed the study (six per dose level and 16 participants on placebo) and were included in the IS and SS. Three participants (two in the 300 mg cohort and one on placebo) had a major deviation and were excluded from PK and PD sets (urine sample at D2 was added into the acid citric container 33 min after collection). All participants were Caucasian male volunteers aged 18 to 50 years (mean = 35.05 ± 10.35 [SD] years) with a BMI ranging from 18.5 to 29.8 kg/m^2^ (mean = 23.64 ± 2.88 [SD] kg/m^2^). The demographics and baseline characteristics are reported in Table 1.

**TABLE 1:** Demographics and baseline characteristics of study participants (safety population^a^).

	DNDI‐6148 10 mg (N = 6)	DNDI‐6148 20 mg (N = 6)	DNDI‐6148 40 mg (N = 6)	DNDI‐6148 80 mg (N = 6)	DNDI‐6148 160 mg (N = 6)	DNDI‐6148 220 mg (N = 6)	DNDI‐6148 300 mg (N = 6)	DNDI‐6148 380 mg (N = 6)	Placebo (N = 16)	Overall (N = 64)
Age (years)										
Mean	35.00	27.00	43.50	39.83	33.33	26.83	33.50	39.00	35.94	35.05
SD	8.32	8.83	7.87	7.73	13.71	10.32	13.37	8.56	8.87	10.35
Median	37.50	23.00	47.50	42.50	33.00	25.00	33.50	39.00	35.00	35.00
Min, max	(21, 43)	(19, 40)	(33, 50)	(28, 48)	(18, 48)	(18, 46)	(18, 50)	(28, 49)	(23, 48)	(18, 50)
Height (cm)										
Mean	172.83	173.33	173.83	177.33	177.17	177.83	180.33	177.67	176.75	176.41
SD	8.21	5.96	4.58	5.79	5.15	5.85	7.31	7.37	5.32	6.11
Median	173.00	172.50	175.00	177.00	176.50	178.00	181.50	176.00	177.00	177.00
Min, max	(162, 183)	(167, 183)	(168, 179)	(170, 184)	(170, 186)	(170, 186)	(167, 188)	(169, 187)	(168, 184)	(162, 188)
Weight (kg)										
Mean	71.12	65.28	73.78	76.15	70.07	68.77	83.63	82.20	72.47	73.52
SD	9.55	4.37	8.90	7.87	2.96	6.41	9.80	9.74	9.87	9.51
Median	69.40	65.35	70.55	78.00	70.70	69.65	84.45	84.05	73.70	72.50
Min, max	(60, 84.5)	(59.9, 72.5)	(65.2, 89)	(65.7, 84.1)	(66.2, 73.2)	(56.9, 75.2)	(70.5, 96.5)	(68.4, 91.8)	(57.6, 86.8)	(56.9, 96.5)
BMI (kg/m^2^)										
Mean	23.82	21.80	24.45	24.25	22.35	21.72	25.78	26.23	23.14	23.64
SD	2.52	2.02	3.08	2.73	0.86	1.28	3.35	4.15	2.62	2.88
Median	23.95	21.65	24.15	23.20	22.65	22.15	26.20	27.55	23.20	23.00
Min, max	(19.7, 27.3)	(18.5, 24.2)	(20.8, 28.2)	(21.7, 29.1)	(21.2, 23.2)	(19.7, 22.8)	(22, 29.8)	(19.8, 29.8)	(19.5, 28.4)	(18.5, 29.8)
Sex, n (%)										
Male	6 (100.0)	6 (100.0)	6 (100.0)	6 (100.0)	6 (100.0)	6 (100.0)	6 (100.0)	6 (100.0)	16 (100.0)	64 (100.0)

^a^ The safety population consisted of all randomized participants who received the study treatment.

### 3.2 Safety and tolerability

A total of 16 AEs were reported in 13 participants (20.3%), of which 13 AEs in 11 participants occurred after treatment initiation and were considered treatment‐emergent AEs (TEAEs) (Table 2). All AEs were either mild (11 cases) or moderate (2 cases) in severity; they resolved by the end of the study, and none led to participant withdrawal. Three TEAEs occurred in 3 participants (18.8%) after placebo administration and 10 TEAEs after any dose of DNDI‐6148 (in 8 participants in total, 14.3%) (Table 2).

**TABLE 2:** Treatment emergent adverse events reported in the safety population^a^ by MedDRA codes (system organ class and preferred terms) ‐ by dose group.

	DNDI‐6148 10 mg (N = 6) participants n (%) [events]	DNDI‐6148 20 mg (N = 6) participants n (%) [events]	DNDI‐6148 40 mg (N = 6) participants n (%) [events]	DNDI‐6148 80 mg (N = 6) participants n (%) [events]	DNDI‐6148 160 mg (N = 6) participants n (%) [events]
**Total TEAEs**	1 (16.7%) [1]	2 (33.3%) [2]	2 (33.3%) [3]	0	1 (16.7%) [1]
**Gastrointestinal disorders**	**0**	**1 (16.7%) [1]**	**1 (16.7%) [1]**	**0**	**0**
Abdominal pain	0	1 (16.7%) [1]	0	0	0
Diarrhoea	0	0	0	0	0
Nausea	0	0	1 (16.7%) [1]	0	0
**General disorders and administration site conditions**	**0**	**0**	**0**	**0**	**0**
Fatigue	0	0	0	0	0
Medical device site reaction	0	0	0	0	0
**Musculoskeletal and connective tissue disorders**	**0**	**1 (16.7%) [1]**	**1 (16.7%) [1]**	**0**	**0**
Arthralgia	0	0	0	0	0
Back pain	0	0	1 (16.7%) [1]	0	0
Pain in extremity	0	1 (16.7%) [1]	0	0	0
**Nervous system disorders**	**1 (16.7%) [1]**	**0**	**1 (16.7%) [1]**	**0**	**1 (16.7%) [1]**
Dizziness postural	0	0	0	0	1 (16.7%) [1]
Headache	1 (16.7%) [1]	0	1 (16.7%) [1]	0	0

	DNDI‐6148 220 mg (N = 6) participants n (%) [events]	DNDI‐6148 300 mg (N = 6) participants n (%) [events]	DNDI‐6148 380 mg (N = 6) participants n (%) [events]	Placebo (N = 16) participants n (%) [events]	Overall (N = 64) participants n (%) [events]
Total TEAEs	0	2 (33.3%) [3]	0	3 (18.8%) [3]	11 (17.2%) [13]
**Gastrointestinal disorders**	**0**	**0**	**0**	**1 (6.3%) [1]**	**3 (4.7%) [3]**
Abdominal pain	0	0	0	0	1 (1.6%) [1]
Diarrhoea	0	0	0	1 (6.3%) [1]	1 (1.6%) [1]
Nausea	0	0	0	0	1 (1.6%) [1]
**General disorders and administration site conditions**	**0**	**1 (16.7%) [1]**	**0**	**1 (6.3%) [1]**	**2 (3.1%) [2]**
Fatigue	0	1 (16.7%) [1]	0	0	1 (1.6%) [1]
Medical device site reaction	0	0	0	1 (6.3%) [1]	1 (1.6%) [1]
**Musculoskeletal and connective tissue disorders**	**0**	**2 (33.3%) [2]**	**0**	**0**	**4 (6.3%) [4]**
Arthralgia	0	2 (33.3%) [2]	0	0	2 (3.1%) [2]
Back pain	0	0	0	0	1 (1.6%) [1]
Pain in extremity	0	0	0	0	1 (1.6%) [1]
**Nervous system disorders**	**0**	**0**	**0**	**1 (6.3%) [1]**	**4 (6.3%) [4]**
Dizziness postural	0	0	0	0	1 (1.6%) [1]
Headache	0	0	0	1 (6.3%) [1]	3 (4.7%) [3]

Abbreviations: AE, adverse event, TEAE: treatment emergent AE.

^a^ The safety population consisted of all randomized participants who received at least one dose of study treatment.

The data represent the number of participants who experienced an AE.

The TEAEs observed were nervous system disorders (four), musculoskeletal and connective tissue disorders (four), gastrointestinal disorders (three) and general disorders and administration site conditions (two). The most reported TEAE in participants receiving DNDI6148 was mild headache (3.6%), and both cases (two participants with one case each) resolved within four hours without the need for treatment. Since DNDI‐6148 crosses the blood–brain barrier, special attention was given to the possible occurrence of even mild neurological events. However, the frequency and severity of headache were not correlated with dose levels.

There were no clinically relevant laboratory evaluation values or vital signs, or physical, psychological or cognitive findings. Plasma CRP and TNFα concentrations in the last four participants of the 160 mg cohort and of the 220–380 mg cohorts were not significantly increased post‐dose, compared with pre‐dose levels. Data are available as Supplementary Information, Text S4.

### 3.3 Pharmacokinetics

**FIGURE 2:**
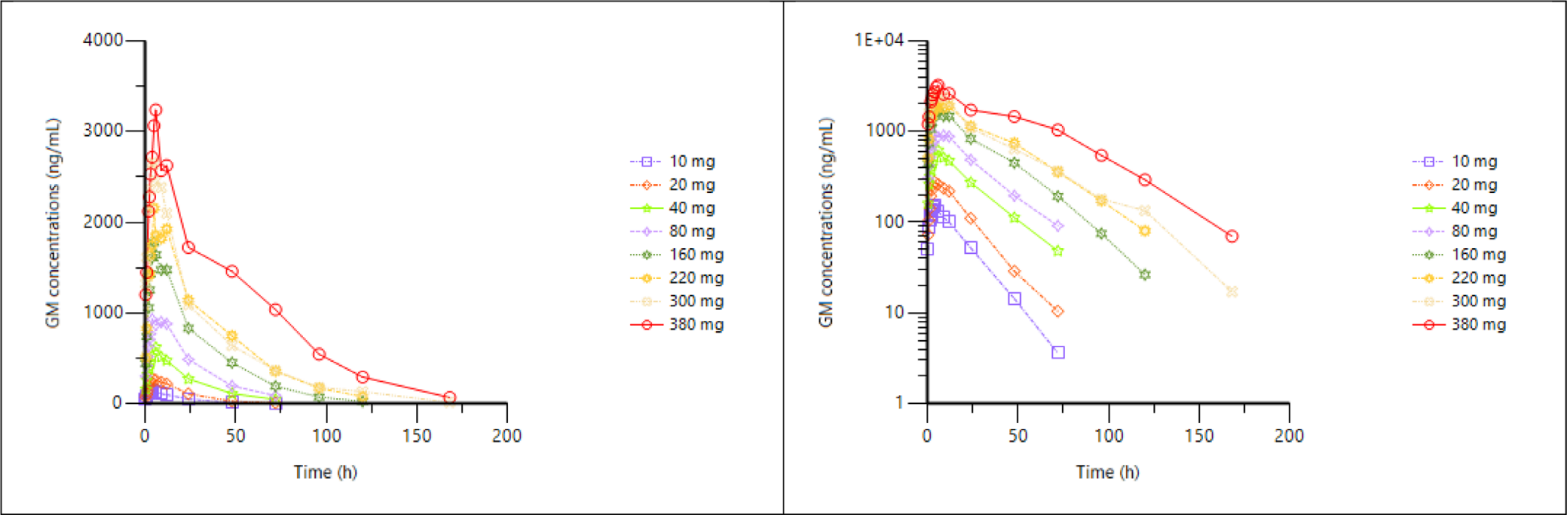
Geometric mean concentrations following treatment with a single dose of DNDI‐6148 are shown on a linear (left) and semi‐logarithmic scale (right). Time of follow‐up increased with doses (10, 20, 40 and 80 mg over 72 h; 160, 220 mg over 120 h and 300 and 380 mg over 168 h). The pharmacokinetic population included all participants who received at least one dose of the IP and for whom pharmacokinetic samples were obtained and analysed.

The geometric mean DNDI‐6148 plasma concentrations over time are shown in Figure 2. The C_max_ and AUC_0–24_ remained below the defined PK‐stopping criteria for all participants. Levels were quantifiable in plasma for up to the last sample collected, i.e. 72 h post‐dose for 10 mg to 80 mg dose levels, 120 h post‐dose for 160 mg and 220 mg dose levels and 168 h post‐dose for 300 mg and 380 mg dose levels.

In general, exposure to DNDI‐6148 (Table 3) increased over the range 10–380 mg of single DNDI‐6148 doses (Table 4). Dose‐proportionality analysis however led to a power estimate of about 0.85 for C_max_ and AUC_0–24_, and around 1 for AUC_0‐∞_. The 90% CIs of the power model for C_max_ and AUC_0–24_ were fully excluded and below the predefined Smith reference interval (0.94–1.06), whereas for AUC_0‐∞_, the lower bound of the 90% CI was included in the reference interval and the upper bound was excluded. Hence, dose‐proportionality could not be accepted, even if the lack of dose‐proportionality was limited for all parameters as the bounds of the 90% CI were close to the reference interval, especially for AUC_0‐∞_. Plasma C_max_ was reached 3 to 12 h after administration and, in most cases, between 4 and 6 h. After this, the decrease in concentrations was multiphasic. T_1/2_ increased with the dose, from 12.80 h to 25.42 h for dosages between 10 and 80 mg DNDI‐6148, then plateaued between 17.47 h and 25.40 h in the 160–380 mg dose range. The CV% for t_1/2_ was, with only a few exceptions, around 25%.

**TABLE 3:** Summary of selected pharmacokinetic parameters in the pharmacokinetic population^a^.

Dose	t_max_^#^	C_max_	AUC_0–24_	AUC_0‐t_	AUC_0‐∞_	t_1/2_	Vd/F	CL/F
**(mg)**	**(h)**	**(ng/mL)**	(h*ng/mL)	(h*ng/mL)	(h*ng/mL)	**(h)**	**(L)**	**(L/h)**	
**10**									
**Mean**	4	156	2′362.51	3′491.04	3′589.96	12.93	53.52	2.97	
**CV%**	3.00–4.00	15.2	18.3	29.6	31.9	21.6	16.7	23.8	
**GM**	‐	155	2′332.72	3′384.53	3′465.56	12.7	52.89	2.89	
**20**									
**Mean**	4	296	4′753.52	7′103.39	7′427.58	14.73	57.82	2.84	
**CV%**	4.00–9.00	17.1	13.4	21.3	24.1	22.9	11.7	27.1	
**GM**	‐	292	4′712.63	6′957.15	7′237.23	14.42	57.51	2.76	
**40**									
**Mean**	5	598	10′564.58	17′952.13	19′781.91	19.91	64.01	2.36	
**CV%**	4.00–6.00	22.2	30.1	35.5	37.4	21.7	33.9	47.5	
**GM**	‐	585	10′132.78	16′864.30	18′416.51	19.52	61.18	2.17	
**80**									
**Mean**	9	1021	18′293.08	33′665.10	42′670.11	25.43	71.15	2.51	
**CV%**	4.00–12.00	14.8	19.4	40.7	57.2	56.7	21.4	56.2	
**GM**	‐	1011	17′982.71	31′186.03	36′859	22.31	69.86	2.17	
**160**									
**Mean**	5	1825.3	31′122.96	62′044.23	63′246.28	18.28	70.79	2.83	
**CV%**	5.00–6.00	11.1	18.4	28.6	29.8	22.9	29.7	44.3	
**GM**	‐	1815.3	30′609.04	59′317.17	60′296.80	17.92	68.6	2.65	
**220**									
**Mean**	5.50	2512.3	41′250.60	91′086.02	96′484.92	24.17	81.32	2.55	
**CV%**	3.00–9.00	44.9	32.6	34.3	40.3	36.1	14.1	33.7	
**GM**		2285.4	39′054.51	87′040.35	90′934.58	23.10	80.62	2.42	
**300**									
**Mean**	5.00	3073.5	48′417.17	104′003.69	105′172.93	23.39	104.45	3.28	
**CV%**	4.00–9.00	32.5	26.9	31.3	31.8	22.6	35.6	51.0	
**GM**	5.12	2959.3	46′860.11	98′231.19	99′174.43	22.91	99.99	3.02	
**380**									
**Mean**	5.50	3606	57′411.25	169′698.06	173′863.53	25.46	79.82	2.26	
**CV%**	4.00–6.00	28.7	13.6	18.6	20.4	26.6	10.5	20.1	
**GM**	5.28	3487.2	56′953.73	167′252.33	170′867.4	24.76	79.44	2.22	

^a^ The pharmacokinetic population included all participants who received the study drug and for whom pharmacokinetic samples were obtained and analysed; ^#^range.

**TABLE 4:** Summary of DNDI‐6148 dose proportionality in the pharmacokinetic population.

Doses (mg)	Dose increase	C_max_	AUC_0–24_	AUC_0‐t_	AUC_0‐oo_
10 to 20	2	1.9	2	2.1	2.1
20 to 40	2	2	2.2	2.4	2.5
40 to 80	2	1.7	1.8	1.8	2
80 to 160	2	1.8	1.7	1.9	1.6
160 to 220	1.375	1.4	1.3	1.5	1.5
220 to 300	1.36	1.2	1.2	1.1	1.1
300 to 380	1.3	1.2	1.2	1.6	1.7

GM of CL/F were similar for all dose levels (between 2 and 3 L/h) with low to high inter‐individual variability. GM of Vd/F ranged from 53 L to 92 L with low to moderate inter‐individual variability.

The percentage of the administered dose recovered in urine as unchanged DNDI‐6148 and the renal clearance were similar for all dose levels, with fe_0–72_ GM values between 0.1% and 0.2% and CLr GM values between 0.003 and 0.004 L/h for dose levels between 40 and 380 mg.

### 3.4 Metabolite profiling and identification of DNDI‐6148 in human plasma

Three putative metabolites of DNDI‐6148 were detected using UHPLC/MS–MS: M1 (oxidative deboronation of DNDI‐6148), M4 (mono‐oxygenation of DNDI‐6148), and M5 (mono‐oxygenation and dehydrogenation of M1). All these metabolites were found in trace amounts.

### 3.5 Cardiac safety

No relevant dose‐dependent increase in the placebo‐ and baseline‐corrected (ΔΔ) QTcF interval was observed after single dose administration of 10 to 380 mg DNDI‐6148. Mean maximum increases varied from 1.5 to 5.5 msec in the DNDI‐6148 dose groups; however, the 95% CI > 10 msec was not found to be of concern since, although concentration‐response modelling indicated a relationship between DNDI‐6148 concentration and ΔΔQTcF, the effects were small and a decrease in ΔΔQTcF was estimated at doses of ≤80 mg. The upper limit of the 90% CI did not exceed 10 msec for any of the single DNDI‐6148 doses, and the maximum effect on ΔΔQTcF for a dose of 380 mg was estimated to be 1.65 (90% CI: −0.635, 3.94) msec.

Combining dose groups, values (90% CI) for ΔΔ heart rate (HR) ranged from −2.9 (−6.3, 0.5) bpm to 5.2 (1.8, 8.5) bpm for DNDI‐6148 and from −3.0 (−6.4, 0.3) bpm to 4.8 (1.4, 8.2) bpm for the placebo, indicating no effect of DNDI‐6148 on HR.

Central tendency analysis did not indicate any effect of DNDI‐6148 on the other ECG parameters, PR interval and QRS duration. Categorical and morphological analyses did not reveal any dose‐dependent effect of DNDI‐6148. No QTcF value >500 msec or change from baseline QTcF >60 msec was recorded in any participant.

Additional cardiac safety analyses using a study‐specific QT correction (QTcG) are provided in Supplementary Information, Text S5 and confirm the absence of a clinically relevant effect of DNDI‐6148 on ΔΔQTcG.

## 4 DISCUSSION

This randomized, placebo‐controlled, SAD trial is the FIH study designed to evaluate the safety and tolerability profile and pharmacokinetic parameters of the benzoxaborole derivative DNDI‐6148 after administration as an oral suspension to healthy male participants.

No safety concerns were identified for DNDI‐6148 over the 10–380 mg dose range evaluated which is anticipated to bracket the daily dose required for efficacy in patients. The product was well tolerated with no clinically relevant findings observed from clinical examination, biological parameters, vital signs or ECG parameters. Circulating levels of CRP and TNFα after dosing were not a cause of concern. The maximal tolerated dose was not reached. No deaths or serious or severe AEs were reported. Since in non‐clinical studies, DNDI‐6148 was shown to cross the blood–brain barrier, special attention was given to neurological events. Although the most reported TEAE in participants receiving DNDi‐6148 was mild headache, the frequency and severity did not correlate with dose and are not considered a cause for concern.

Results further indicated that single administration of doses up to 380 mg DNDI‐6148 did not cause a relevant increase in QTcF or affect the PR interval, QRS duration or HR.

Plasma concentrations of DNDI‐6148 had similar patterns across all dose levels and increased with dose to reach their peak, generally with a median t_max_ of 4–6 h. Plasma C_max_ and AUC increased up to 380 mg, but dose‐proportionality tested using a power model statistical approach was not formally demonstrated, with a power estimate around 0.85 for C_max_ and AUC_0–24_, and around 1 for AUC_0‐∞_. The GM half‐life in plasma was not constant across dose levels, being around 13 h for 10 and 20 mg doses and increasing to around 20 h for the higher doses. Apparent clearance was similar for all dose levels. The apparent volume of distribution suggested a moderate tissue distribution, with low to moderate inter‐individual variability and increase with doses, which may contribute to the observed increase in the half‐life in plasma. However, other factors could also explain the observed trends in half‐life, such as solubility‐limited absorption along with potential effects on apparent clearance (CL/F). These factors will be further examined in ongoing population PK modelling (manuscript in preparation). Further studies, including mass balance, are required to fully characterize the PK profile of DNDI‐6148.

The percentage of the administered dose recovered in urine as unchanged DNDI‐6148 (0.1% to 0.2% of the dose) and the renal clearance were low and similar between 40 and 380 mg doses. At the highest dose tested (380 mg), exploratory metabolic profiling of DNDI‐6148 in plasma detected and identified three putative metabolites corresponding to oxidative deboronation and to mono‐oxygenation of DNDI‐6148 and to mono‐oxygenation and dehydrogenation of the deboronated metabolite, as suggested from in vitro experiments in liver microsomes.^9^ These metabolites were detected only as traces in plasma, suggesting slow metabolism by the liver. These results are in line with in vitro data that demonstrated that DNDI‐6148 (at 1 μM) is cleared slowly in human liver microsomes (CL_int_ below 0.3 μL/min/mg) or in hepatocytes (CL_int_ of 0.5 μL/min/10^6^ cells). They are also in line with the PK, metabolism and excretion of acoziborole, another derivative of the same chemical class, for which a slow liver metabolism and predominant hepato‐biliary elimination were reported in humans.^15^, ^16^

In summary, DNDI‐6148 distribution was moderate, consistent with its low‐to‐moderate lipophilicity (logD 1.92 at pH 7.4) and plasma protein binding (over 93% bound). Clearance was low in both preclinical species and the present study, with minimal renal excretion (<0.2% unchanged drug in urine) and limited metabolism. Slow hepatic metabolism with minor metabolite formation was observed in vitro (liver microsomes and hepatocytes) and confirmed by detection of only trace metabolites in this study. The exposure, clearance, distribution and half‐life values for DNDI‐6148 indicate that this molecule would be appropriate for further clinical development; additionally, single oral doses from 10 mg to 380 mg were well tolerated and the data support further investigation with multiple dosing.

## AUTHOR CONTRIBUTIONS

J.‐Y. G., S. Bl., B. A. and Y. D. contributed to the study design. J.‐Y. G., S. D., D. L., S. Bl., P. M., S. L., B. A., M. L‐B. and Y. D. performed the study research. All authors analysed the data. J.‐Y. G. wrote the manuscript. All authors were involved in revising the manuscript and approved the final manuscript.

## CONFLICT OF INTEREST STATEMENT

The authors are employed by their respective organizations and declare no conflicts of interest.

## Supporting information


**Figure S1.** Relationship between QT and RR intervals under drug‐free conditions in healthy participants.
**Figure S2. Changes in QTcG intervals (group‐corrected) over time, by dose**. Changes from baseline (ΔQTcG) were calculated as the difference between post‐dose and pre‐dose QTcG for each subject and time point. Placebo‐corrected changes (ΔΔQTcG) were calculated as the difference between each subject's ΔQTcG value and the time‐matched mean ΔQTcG in the placebo group. Bars represent the mean ± 90% CI. The red dashed line indicates the 10‐msec threshold for QTcG prolongation; the grey dashed line represents the zero line.
**Figure S3. Changes in heart rate over time, by dose**. ΔHR was calculated as the difference between post‐dose and pre‐dose HR for each subject and time point. ΔΔHR was calculated as the difference between each subject's ΔHR and the time‐matched mean ΔHR in the placebo group. Bars represent the mean ± 90% CI. The grey dashed line represents the zero line.
**Figure S4.** Relationship between observed DNDI‐6148 plasma concentration and its effects on **a)** ΔΔQTcG and **b)** ΔΔHR in healthy participants receiving single oral doses ranging from 10 to 380 mg. Observed ΔΔQTcG and ΔΔHR values are shown as points. The solid blue lines represent the regression fit from ordinary linear regression, with shaded areas depicting the 90% confidence intervals around the predicted mean values. The black dashed line represents the zero line, and the red dashed line in panel a) indicates the 10 msec threshold for QTcG prolongation. Grey vertical shaded areas correspond to the arithmetic mean C_max_ levels in each dose group (p‐values for slope in brackets).

## Data Availability

The data underlying the results of this study are available upon request because they contain potentially sensitive personal information, which must be de‐identified at the individual level. Interested researchers may request access to de‐identified participant data from Vivli, the data‐sharing partner of the Drugs for Neglected Diseases initiative (DNDi), commissioner of this study, at https://vivli.org/ourmember/dndi/.
