# Supplementary material for: Safety, tolerability and pharmacokinetics of DNDI‐6148, a novel agent for leishmaniasis: A randomized, controlled, single ascending dose study in healthy participants
Source: Br J Clin Pharmacol. 2025 Nov 20;92(4):1087–97. doi: 10.1002/bcp.70328 (PMC13021292; doi:10.1002/bcp.70328)
Supplement: Supplementary file 1 — Figure S1. Relationship between QT and RR intervals under drug‐free conditions in healthy participants. Figure S2. Changes in QTcG intervals (group‐corrected) over time, by dose. Changes from baseline (ΔQTcG) were calculated as the difference between post‐dose and pre‐dose QTcG for each subject and time point. Placebo‐corrected changes (ΔΔQTcG) were calculated as the difference between each subject's ΔQTcG value and the time‐matched mean ΔQTcG in the placebo group. Bars represent the mean ± 90% CI. The red dashed line indicates the 10‐msec threshold for QTcG prolongation; the grey dashed line represents the zero line. Figure S3. Changes in heart rate over time, by dose. ΔHR was calculated as the difference between post‐dose and pre‐dose HR for each subject and time point. ΔΔHR was calculated as the difference between each subject's ΔHR and the time‐matched mean ΔHR in the placebo group. Bars represent the mean ± 90% CI. The grey dashed line represents the zero line. Figure S4. Relationship between observed DNDI‐6148 plasma concentration and its effects on a) ΔΔQTcG and b) ΔΔHR in healthy participants receiving single oral doses ranging from 10 to 380 mg. Observed ΔΔQTcG and ΔΔHR values are shown as points. The solid blue lines represent the regression fit from ordinary linear regression, with shaded areas depicting the 90% confidence intervals around the predicted mean values. The black dashed line represents the zero line, and the red dashed line in panel a) indicates the 10 msec threshold for QTcG prolongation. Grey vertical shaded areas correspond to the arithmetic mean Cmax levels in each dose group (p‐values for slope in brackets). [file BCP-92-1087-s001.docx]

**SUPPLEMENTARY INFORMATION**

**Supplementary Text S1: Inclusion / exclusion criteria**

**1. Subject inclusion criteria**

1. Healthy Caucasian male subject aged 18 to 50 years inclusive,
2. Non-smoker subject or light smoker of not more than 5 cigarettes a day. No smoking (or use of smoking substitute e.g. nicotine patch) was permitted from screening throughout the study,
3. Body Mass Index (BMI) between 18 and 30.1 kg/m^2^ inclusive at screening,
4. Considered as healthy after a comprehensive clinical assessment (detailed medical history and complete physical and neurological examination),
5. Normal Blood Pressure (BP) and Heart Rate (HR) at the screening visit after 10 minutes in supine position:

- 95 mmHg ≤ Systolic Blood Pressure (SBP) ≤ 140 mmHg,
- 50 mmHg ≤ Diastolic Blood Pressure (DBP) ≤ 90 mmHg,
- 45 bpm ≤ HR ≤ 90 bpm,
- or considered NCS by investigators,

1. Normal ECG recording on a 12-lead ECG at the screening visit:

- 120 ms < PR < 210 ms,
- QRS < 120 ms,
- QTcF ≤ 430 ms for male,
- No sign of any relevant trouble of sinusal automatism,
- or considered as non-clinically significant by investigators,

1. Laboratory parameters within the normal range of the laboratory (haematological, hormonology, blood chemistry tests, urinalysis). Individual values out of the normal range could be accepted if judged non-clinically significant by the Investigator,
2. ALAT, ASAT and creatinine values strictly within the normal range,
3. A negative result for diagnostic test of SARS-CoV-2 at day D-1,
4. Normal dietary habits,
5. Provision of written informed consent to participate as shown by a signature on the volunteer consent form, after reading the information and consent form and after having the opportunity to discuss the trial with the investigator or his delegate,
6. Able to communicate well with the Investigator and research staff and to comply with the requirements of the entire study,
7. Covered by Health Insurance System and / or in compliance with the recommendations of National Law in force relating to biomedical research,
8. Agreeing to adhere to the contraception requirements: use of condom by the male subject plus an effective method of contraception for the subject or the subject partner of childbearing potential from study drug administration until 90 days post-dosing or use of a condom for 10 days post-dosing if the partner was known to be pregnant.

**2. Subject exclusion criteria**

1. Participation in another clinical trial within 3 months prior and during the study, or 5‐times the half‐life of the drug tested in the previous clinical trial, whichever was longer (time calculated relative to the last dose in the previous clinical trial),
2. Any history or presence of cardiovascular, pulmonary, gastro-intestinal, hepatic, renal, metabolic, haematological, neurologic, psychiatric, systemic or infectious acute or chronic disease; including known or suspected HIV, HBV or HCV infection,
3. With any clinically significant abnormality following review of pre‐study laboratory tests, vital signs, full physical examination, and ECG,
4. Symptomatic hypotension whatever the decrease of blood pressure or asymptomatic postural hypotension defined by a decrease in SBP or DBP equal to or greater than 20 mmHg within two minutes when changing from the supine to the standing position,
5. History of allergy, intolerance or photosensitivity to any drug,
6. History of serious allergy, asthma, allergic skin rash or sensitivity to any drug,
7. History of additional risk factors for “Torsades de Pointe” (e.g., heart failure, hypokalemia, family history of Long QT Syndrome),
8. Current suicide risk or history of suicide risk (CSSRS baseline: “yes” answer to items 4 and/or 5); subjects with a “yes” answer for current suicide risk should be referred for psychiatric evaluation,
9. Subjects with rare hereditary problems of fructose intolerance, glucose‐galactose malabsorption or sucrase‐isomaltase insufficiency,
10. Use of a prescription medicine during the 28 days before the first dose of trial medication or use of an over‐the‐counter medicine (including antacid drug, except for acetaminophen (paracetamol)), during the 7 days before the first dose of trial medication,
11. History or presence of drug or alcohol abuse (more than 14 units of alcohol per week, one unit = 8 g or about 10 mL of pure alcohol),
12. Excessive consumption (more than one liter / day) of beverages with xanthine bases,
13. Consumption of more than 8 cups daily of beverage containing caffeine,
14. Regular daily consumption of more than 5 cigarettes daily, or use more than 3 grams (1/8 ounce) of tobacco,
15. Use of dietary supplements or herbal remedies (such as St John’s Wort) known to interfere with the CYP3A4 and/or P‐gp metabolic pathways during the 28 days before the first dose of trial medication. Grapefruit was also to be avoided during the 7 days before the first dose of trial medication,
16. Positive Hepatitis B surface (HBs) antigen or anti Hepatitis C Virus (HCV) antibody, or positive results for Human Immunodeficiency Virus (HIV 1 or 2) tests,
17. Positive results of screening for drugs of abuse (opiates, cocaine, amphetamine, cannabis, benzodiazepines),
18. Blood donation (including in the frame of a clinical trial) within 12 weeks before administration,
19. General anaesthesia within 3 months before trial medication administration,
20. Inability to abstain from intensive muscular effort,
21. Any clinical condition or prior therapy which, in the opinion, of the Investigator, made the subject unsuitable for the study,
22. Surgery (e.g. stomach bypass) or medical condition that might affect absorption of study drug taken orally,
23. Febrile illness within 1 week before the start of the study,
24. Subject who, in the judgment of the Investigator, was likely to be non-compliant or uncooperative during the study, or unable to cooperate,
25. No possibility of contact in case of emergency,
26. Exclusion period of a previous study,
27. Administrative or legal supervision,
28. Unwillingness to give their informed consent,
29. Subject having received indemnities above a certain threshold for participation in biomedical research within the 12 last months, including the indemnities for the present study.

**Supplementary Text S2: Stopping rules**

**1. Trial stopping criteria**

- a ‘serious’ adverse reaction (SAR) (i.e. a serious adverse event (SAE) considered at least possibly related to DNDI‐6148) occurring in one subject or,
- ‘severe’ non‐serious adverse reactions (AR) (i.e. severe non‐serious adverse events considered as, at least, possibly related to DNDI‐6148) occurring in two subjects in the same cohort, independent of within or not within the same system organ class (SOC).

**2. Stopping criteria within a cohort**

- 2 subjects experiencing severe non-serious AEs considered as at least possibly related to the study drug,
- 2 subjects experiencing a significant increase (i.e. > 5 upper limit of normal value [ULN]) of ALAT.

**3. Dose escalation stopping criteria between cohorts**

- 2 subjects of the same cohort experiencing:
  - a significant increase of ALAT > 3 ULN or,
  - simultaneous increases of total bilirubin > 2 ULN, ALAT > 2 ULN and alkaline phosphatases > 1.5 ULN or,
- 6 subjects of the same cohort experiencing study drug related moderate, non-serious AEs (that are considered at least possibly related to the drug effect i.e. moderate, non-serious ARs) or,
- 1 subject of the cohort presenting a C_max_ or AUC_0-24_ value above corresponding plasma exposure at no adverse effect level [NOAEL] in NHP

**Supplementary Text S3: Bioanalytical methods used for quantification and identification of DNDI-6148 metabolites**

Metabolic profiling was conducted using plasma samples from the highest dosing cohort (380 mg DNDi-6148) collected at 0, 4, 9, and 24 hours post-dose. Samples were stored below -65°C until analysis at SGS Life Sciences (Wavre, Belgium).

Unacidified plasma samples were pooled by time point for primary analysis. Acidified plasma samples (300 µL per sample; EDTA plasma with H₃PO₄, 2%, v/v, 50:50) were pooled and analyzed under identical conditions to confirm the absence of degradation artifacts during sample handling.

For analysis, 0.5 mL of pooled unacidified plasma or 1 mL of pooled acidified plasma was spiked with 10 µL of internal standard and subjected to protein precipitation with 3 mL of acetonitrile containing 0.1% formic acid. After vortex mixing and centrifugation (4000 rpm, 15 minutes, 4°C), the supernatants were transferred to glass tubes and evaporated to dryness under a stream of nitrogen at 40°C. The residues were reconstituted in 120 µL of injection solvent and 20 µL was transferred to a 96-well plate and diluted with 380 µL of injection solvent. Following vortex and centrifugation (4000 rpm, 1 minute, room temperature), a 5 µL aliquot was injected into the UHPLC/MS-MS system.

UHPLC/MS-MS analysis was performed using an Agilent 1290 UHPLC system coupled to an AB/MDS API 4000-Q-trap mass spectrometer. Chromatographic separation was achieved using an Acquity HSS T3 column (1.8 µm, 100 × 2.1 mm) at 40°C. The mobile phase consisted of high-purity water with 0.1% formic acid (mobile phase A) and acetonitrile with 0.1% formic acid (mobile phase B). The flow rate was 0.5 mL/min. The gradient program started with 100% mobile phase A, decreased to 70% at 8 minutes, 35% at 16 minutes, and 20% at 18 minutes, then returned to 100% mobile phase A at 18.1 minutes and holding until 20 minutes.

Detection began with EMS (Enhanced Mass Scan) and EPI (Enhanced Product Ion) modes to investigate the presence of all putative metabolites of DNDi-6148. Samples were then reinjected to confirm the presence of observed metabolites using MRM (Multiple Reaction Monitoring) mode. The following transitions were monitored: DNDi-6148 (336.2 → 308.1 Da), M1 (326.0 → 308.0 Da), M4 (352.0 → 306.0 Da), and M5 (340.0 → 322.0 Da).

**Supplementary Text S4: C-reactive protein (CRP) plasma concentrations pre- (-1 h) and post- (6 h) dose in the safety population (cohorts treated with 160 – 380 mg DNDI-6148)**

**Value Change from Pre-dose**

**Cohort Visit n Mean Min/Median Mean Min/Median**

**± SD /Max ± SD /Max**

DNDI-6148 160 mg D1-PRE-DOSE 3 0.83 ± 0.60/0.60 - -

(N=6) 0.40 /1.30

D1-T6H 3 0.80 ± 0.60/0.60 -0.03 ± -0.10/0.00

0.35 /1.20 0.06 /0.00

DNDI-6148 220 mg D1-PRE-DOSE 6 0.75 ± 0.60/0.60 - -

(N=6) 0.37 /1.50

D1-T6H 6 0.72 ± 0.60/0.60 -0.03 ± -0.20/0.00

0.29 /1.30 0.08 /0.00

DNDI-6148 300 mg D1-PRE-DOSE 6 2.60 ± 0.60/1.30 - -

(N=6) 3.57 /9.80

D1-T6H 6 2.47 ± 0.60/1.15 -0.13 ± -0.30/-0.15

3.52 /9.60 0.12 /0.00

DNDI-6148 380 mg D1-PRE-DOSE 6 0.88 ± 0.60/0.60 - -

(N=6) 0.65 /2.20

D1-T6H 6 0.87 ± 0.60/0.60 -0.02 ± -0.10/0.00

0.61 /2.10 0.04 /0.00

Placebo (N=8) D1-PRE-DOSE 7 2.91 ± 0.60/0.80 - -

4.38 /12.50

D1-T6H 7 2.80 ± 0.60/1.00 -0.11 ± -0.60/0.00

4.29 /12.30 0.29 /0.30

**Tumour necrosis factor (TNFα) plasma concentrations pre- (-1 h) and post- (6 h) dose in the safety population (cohorts treated with 160 – 380 mg DNDI-6148)**

**Value Change from Pre-dose**

**Cohort Visit n Mean Min/Median Mean Min/Median**

**± SD /Max ± SD /Max**

DNDI-6148 160 mg D1-PRE-DOSE 3 2.40 ± 1.68/1.73 - -

(N=6) 1.21 /3.80

D1-T6H 3 2.55 ± 2.35/2.58 0.15 ± -1.45/0.090

0.19 /2.73 1.39 /1.00

DNDI-6148 220 mg D1-PRE-DOSE 6 21.45 ± 3.92/17.15 - -

(N=6) 18.25 /56.78

D1-T6H 6 1.77 ± 0.00/1.74 -19.68 ± -55.63/-15.26

1.25 /3.79 18.54 /-2.21

DNDI-6148 300 mg D1-PRE-DOSE 5 3.04 ± 1.36/2.34 - -

(N=6) 1.93 /6.30

D1-T6H 6 2.67 ± 1.54/2.62 -0.37 ± -2.68/0.18

0.73 /3.62 1.40 /0.93

DNDI-6148 380 mg D1-PRE-DOSE 5 2.69 ± 2.21/2.56 - -

(N=6) 0.47 /3.47

D1-T6H 4 2.82 ± 2.58/2.75 0.33 ± 0.14/0.32

0.28 /3.22 0.16 /0.52

Placebo (N=8) D1-PRE-DOSE 5 6.26 ± 1.58/2.58 - -

8.99 /22.31

D1-T6H 6 2.36 ± 1.06/2.62 -3.94 ± -19.04/-0.69

0.99 /3.35 8.54 /1.77

**Supplementary Text S5: Additional Cardiac safety analysis**

Triplicate ECGs extracted from 24-hour Holter recordings at predefined time points aligned with the pharmacokinetic (PK) sampling were used for additional cardiac safety analyses. QT intervals were corrected for variations in heart rate using Bazett’s (𝛼 = 1/2) and Fridericia’s (𝛼 = 1/3) methods, and a study-specific group correction method (𝛼 = 0.385) according to the formula: $QTcG={QT}/{{RR}^{\alpha}}$. For the group correction, α was estimated as the slope of the linear relationship between ln(QT) and ln(RR), based on drug-free (placebo and pre-dose) data (**Figure S1**). The adequacy of the different correction methods was assessed by linear regression of QTc vs RR, which showed no significant trend (p = 0.807) in drug-free data for the group correction (QTcG), representing an advantage over Bazett’s and Fridericia’s corrections.

| 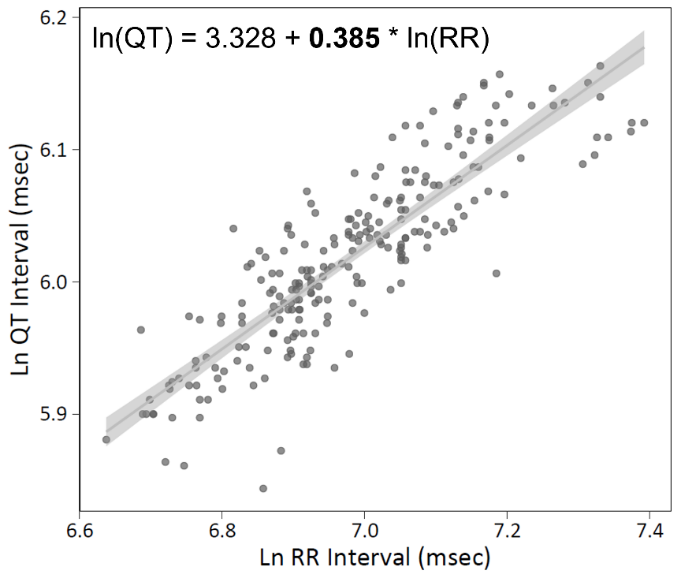 | **Fig S1.** **Relationship between QT and RR intervals under drug-free conditions in healthy participants.**  The estimated slope (0.385) from linear regression was used as a study-specific correction factor to derive QTcG (group-wise corrected QT interval). |
| --- | --- |

**Central tendency analysis at baseline**

At baseline, the median QTcG was 403 msec (min–max: 383–430) in the placebo group, with similar values across DNDI-6148 dose groups (medians: 387–417 msec; min–max: 352–428). The median heart rate was 54 beats/min (min–max: 40–75) in the placebo group and ranged from 55 to 63 beats/min (min–max: 46–73) across dose groups.

**The effect of DNDI-6148 on QTcG and HR**

Changes from baseline (ΔQTcG, ΔHR) and placebo-corrected changes (ΔΔQTcG, ΔΔHR), adjusted for circadian rhythm, are shown by time and dose level in **Figures S2 and S3**. Following single-dose administration of DNDI-6148, no relevant increase in ΔΔQTcG was observed. The largest mean ΔΔQTcG was 7.24 msec (90% CI: 4.22, 10.25) at 12 h post-dose with 160 mg. Isolated instances where the upper bound of the one-sided 95% CI exceeded 10 msec were observed at lower doses (10–40 mg), but not at 80, 220, or 300 mg. In the 380 mg group, only one time point exceeded the threshold (1.83 msec; 90% CI: -7.2, 10.86) at 9 h post-dose. No clear temporal pattern or alignment with C_max_ (T_max_: ~4–6 hours) was observed, suggesting no dose-dependent effect of DNDI-6148 on QTcG. Moreover, no QTcG values exceeded 450 msec, nor were any changes from baseline greater than 30 msec. Similarly, no relevant, dose-dependent increase in ΔΔHR was observed.


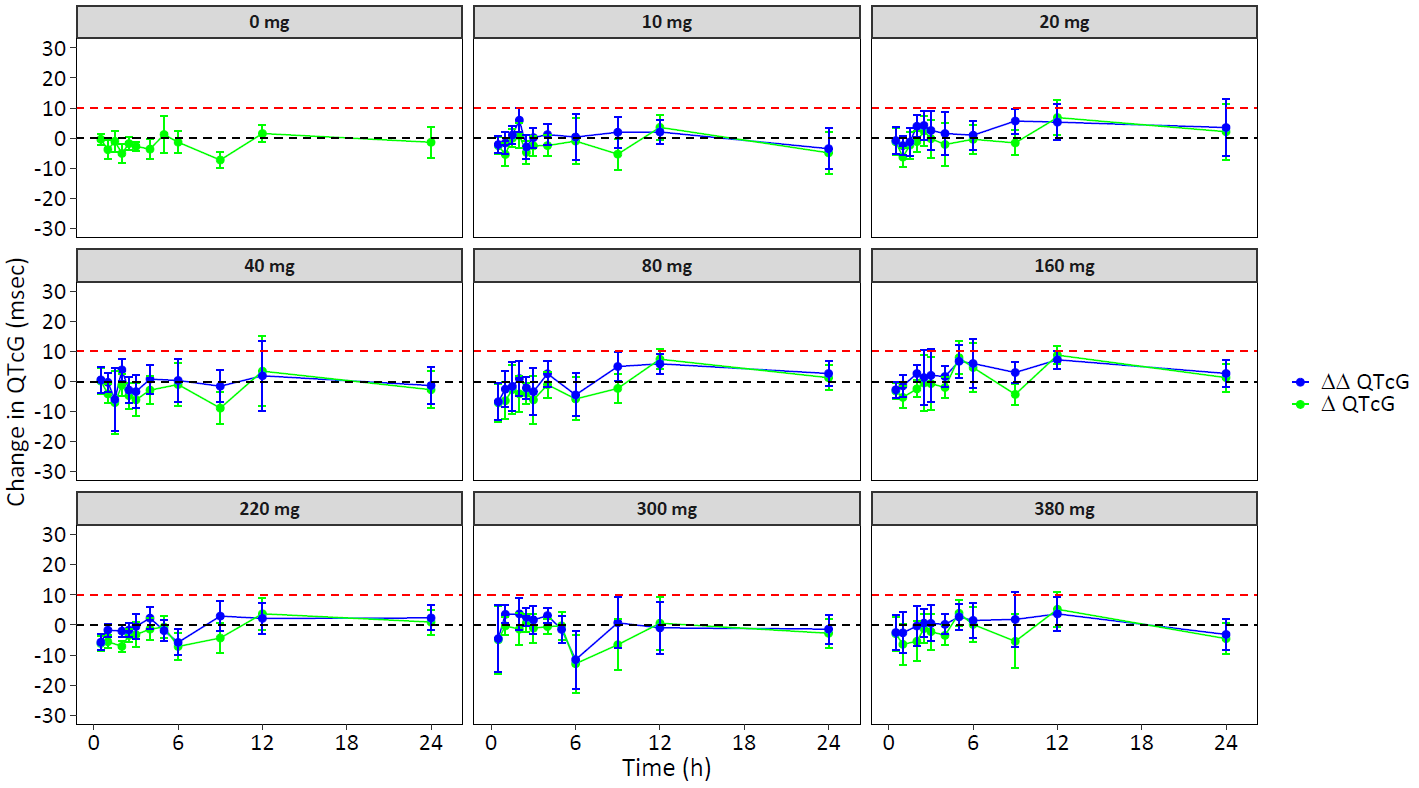
**Fig S2.** **Changes in QTcG intervals (group-corrected) over time, by dose.** Changes from baseline (ΔQTcG) were calculated as the difference between post-dose and pre-dose QTcG for each subject and time point. Placebo-corrected changes (ΔΔQTcG) were calculated as the difference between each subject’s ΔQTcG value and the time-matched mean ΔQTcG in the placebo group. Bars represent the mean ± 90% CI. The red dashed line indicates the 10-msec threshold for QTcG prolongation; the grey dashed line represents the zero line.


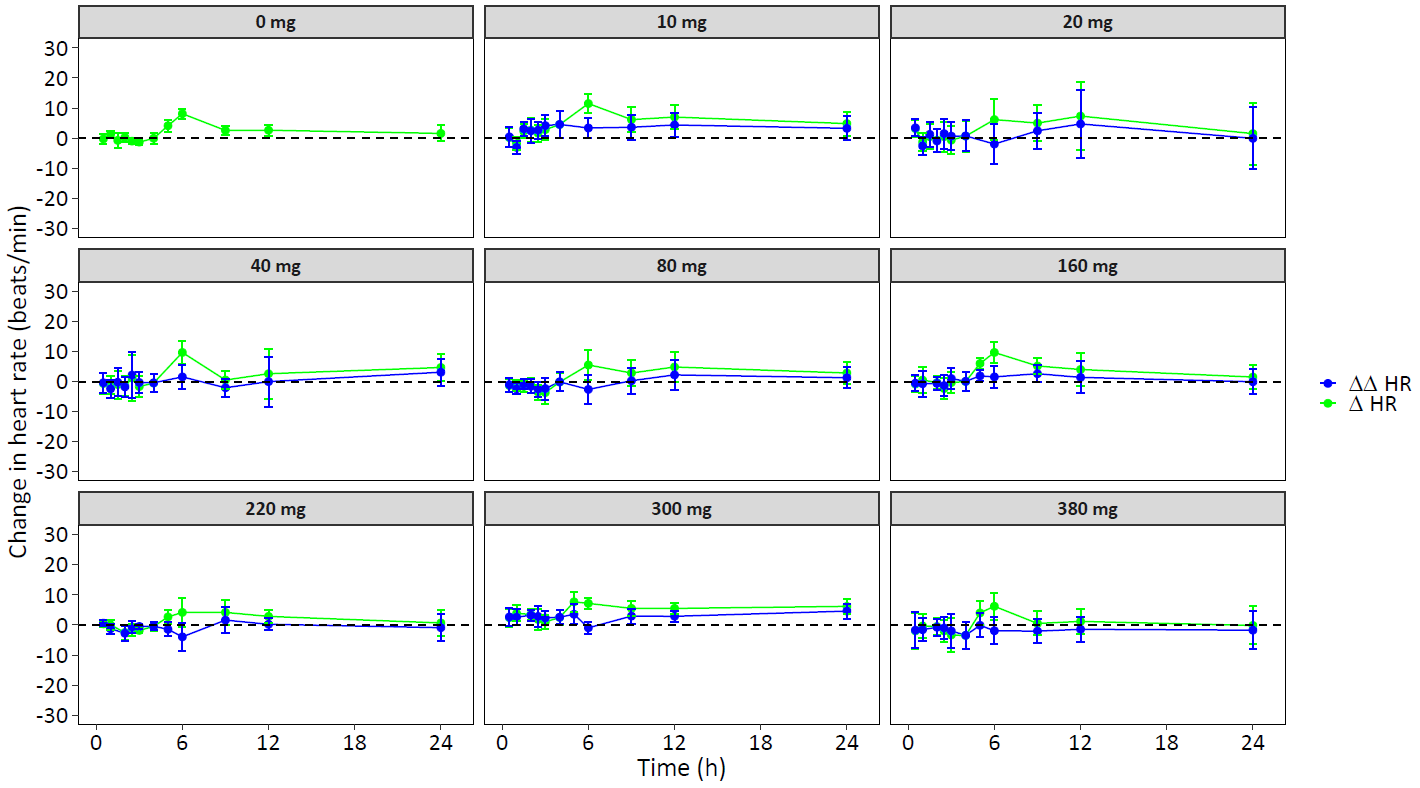


**Fig S3.** **Changes in heart rate over time, by dose.** ΔHR was calculated as the difference between post-dose and pre-dose HR for each subject and time point. ΔΔHR was calculated as the difference between each subject’s ΔHR and the time-matched mean ΔHR in the placebo group. Bars represent the mean ± 90% CI. The grey dashed line represents the zero line.

**Concentration - response analysis**

Linear regression confirmed these findings, showing no significant relationship between plasma DNDI-6148 concentrations and ΔΔQTcG (p=0.163; **Figure S4a**). The 90% CI of the regression line did not exceed the 10 msec threshold, even at C_max_ in the highest dosing cohort. Similarly, no significant correlation was found for ΔΔHR (p = 0.425; **Figure S4b**).


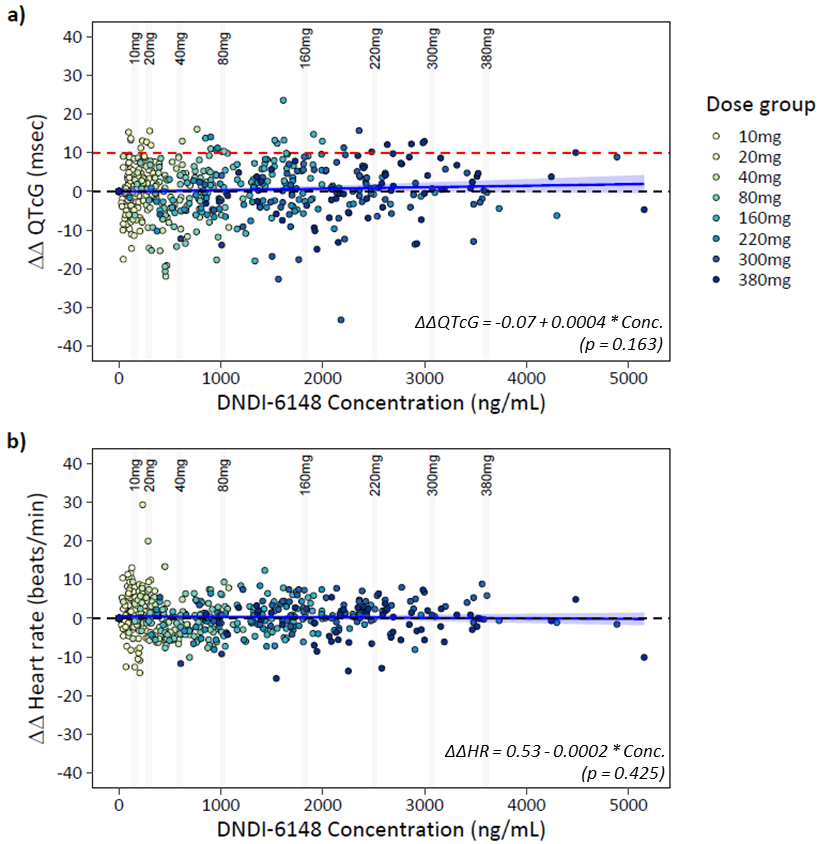


**Fig S4.** Relationship between observed DNDI-6148 plasma concentration and its effects on **a)** ΔΔQTcG and **b)** ΔΔHR in healthy participants receiving single oral doses ranging from 10 to 380 mg. Observed ΔΔQTcG and ΔΔHR values are shown as points. The solid blue lines represent the regression fit from ordinary linear regression, with shaded areas depicting the 90% confidence intervals around the predicted mean values. The black dashed line represents the zero line, and the red dashed line in panel a) indicates the 10 msec threshold for QTcG prolongation. Grey vertical shaded areas correspond to the arithmetic mean C_max_ levels in each dose group (p-values for slope in brackets).
